# Investigation of the Effect of PbO Doping on Telluride Glass Ceramics as a Potential Material for Gamma Radiation Shielding

**DOI:** 10.3390/ma16062366

**Published:** 2023-03-15

**Authors:** Artem L. Kozlovskiy, Dmitriy I. Shlimas, Maxim V. Zdorovets, Edgars Elsts, Marina Konuhova, Anatoli I. Popov

**Affiliations:** 1 Engineering Profile Laboratory, L.N. Gumilyov Eurasian National University, Satpaev Str. 5, Astana 010008, Kazakhstan; 2 Institute of Geology and Oil and Gas Business, Satbayev University, Satbayev St. 22, Almaty 050032, Kazakhstan; 3 Institute of Solid State Physics, University of Latvia, 8 Kengaraga Str., LV-1063 Riga, Latvia; 4 Engineering Research Institute, “Ventspils International Radio Astronomy Centre”, Ventspils University of Applied Sciences, 101 Inzenieru Str., LV-3601 Ventspils, Latvia

**Keywords:** shielding materials, doping, absorption, optical properties, protective materials

## Abstract

The purpose of this paper is to study the effect of PbO doping of multicomponent composite glass-like ceramics based on TeO_2_, WO_3_, Bi_2_O_3_, MoO_3_, and SiO_2_, which are one of the promising materials for gamma radiation shielding. According to X-ray diffraction data, it was found that the PbO dopant concentration increase from 0.10 to 0.20–0.25 mol results in the initialization of the phase transformation and structural ordering processes, which are expressed in the formation of SiO_2_ and PbWO_4_ phases, and the crystallinity degree growth. An analysis of the optical properties showed that a change in the ratio of the contributions of the amorphous and ordered fractions leads to the optical density increase and the band gap alteration, as well as a variation in the optical characteristics. During the study of the strength and mechanical properties of the synthesized ceramics, depending on the dopant concentration, it was found that when inclusions in the form of PbWO_4_ are formed in the structure, the strength characteristics increase by 70–80% compared to the initial data, which indicates the doping efficiency and a rise in the mechanical strength of ceramics to external influences. During evaluation of the shielding protective characteristics of the synthesized ceramics, it was revealed that the formation of PbWO_4_ in the structure results in a rise in the high-energy gamma ray absorption efficiency.

## 1. Introduction

Today, there is a growing focus on the use of ionizing radiation sources in various fields, especially in the field of medical applications and nuclear energy [1,2,3]. Extensive use of ionizing radiation sources requires not only their constant monitoring, but also the search for alternative solutions for effective protection against radiation’s adverse effects on living organisms and electronics [4,5]. The adopted international program ALARA [6,7], aimed to control the use and reduction of the risks of ionizing radiation negative effects, also includes several measures related to the development of new methods for protecting and shielding ionizing radiation and improving existing protection technologies by increasing the radiation absorption efficiency. At the same time, neutron and gamma radiation, which, by virtue of their nature, have a high penetrating ability and can cause detrimental effects in the case of irradiation of materials and living organisms, present the greatest harm from all known types of radiation [8,9,10].

Classic methods of protection against ionizing radiation, which has a high penetrating ability, is the use of materials that can effectively absorb radiation and reduce its intensity. At the same time, this method uses such techniques as increasing protective material thickness, if possible, and using complex multicomponent materials [11,12,13]. If the thickness increases, everything is quite simple: the thicker the obstacle in the path of radiation, the less likely it is to pass through the material. However, this method is effective only in the case of building large fundamental plants, such as nuclear power plants or nuclear research reactors for protection against neutron radiation. At the same time, in most cases, increasing the thickness is not an optimal and effective way, and if it is necessary to create local protection, the thickness of the protective materials can only have a negative effect, in view of the need to maintain the overall dimensions and parameters of the installations [14,15]. In this case, as a rule, the second protection method is used: protective material composition variation to obtain optimal characteristics with the same weight and overall dimensions and parameters. Great prospects in this direction are shown by materials, such as glasses or glass-like ceramics obtained from a mixture of oxide compounds or rare earth elements. So, for example, one of the leading experts in this field, Professor M.I. Sayyed, in the last several years, has proposed a number of different compositions of glasses and ceramics based on compounds such as TeO_2_, Bi_2_O_3_, WO_3_, ZnO, NaO, BO, CeO_2_, MoO_3_, SiO_2_, etc. [16,17,18,19,20]. According to the data presented in several works [17,18,19,20,21,22,23,24], based on the combination of all the results obtained, one might conclude that the shielding characteristics strongly depend on the number of glass components and their concentration, which affects the value of the linear and mass absorption coefficient, which is one of the most important parameters in gamma and neutron radiation shielding. Also, important characteristics in shielding materials are the parameters of resistance to external influences, including mechanical or long-term radiation exposure, which has a cumulative effect. All this, despite the large number of studies in this area, requires new research and experimental confirmation of the efficiency of shielding of new types of materials depending on their composition and applications [25,26,27,28,29,30,31,32,33,34,35].

Based on the above, the purpose of this study is to study the variation of the components in glass-like ceramics of the (1 − x)TeO_2_ − 0.2WO_3_ − 0.1Bi_2_O_3_ − 0.1MoO_3_ − 0.1SiO_2_ − xPbO type on the gamma radiation shielding efficiency. The choice of the components of oxide compounds is based on their physicochemical, optical, and structural characteristics, the combination of which makes it possible to create multicomponent glass-like ceramics with a high gamma-ray shielding efficiency. In this case, the use of the PbO compound as one of the glass components to replace TeO_2_ is due to the possibility of the shielding efficiency growth by increasing the density of glass-like ceramics, while maintaining their optical properties [36,37,38]. In several works [37,38,39], it was shown that the use of PbO as a dopant is a very promising way to increase the shielding efficiency and the stability of shielding materials [39,40].

## 2. Experimental Part

For the synthesis of the studied objects, chemical compounds based on TeO_2_, WO_3_, Bi_2_O_3_, MoO_3_, SiO_2_, AND PbO were chosen in the form of powders with a particle size of at least 1–10 μm with the chemical purity of 99.95%. All initial powders were purchased from Sigma Aldrich (Sigma Aldrich, St. Louis, MO, USA). Multicomponent composite glass-like ceramics of the (1 − x)TeO_2_ − 0.2WO_3_ − 0.1Bi_2_O_3_ − 0.1MoO_3_ − 0.1SiO_2_ − xPbO type were chosen as the objects of study. The choice of ceramic components is due to the combination of their physicochemical, strength and optical properties, which make it possible to create the most effective material for shielding gamma radiation. The choice of TeO_2_ AND WO_3_ compounds as the main components of ceramics is due to their prospects for use as alternative materials for creating protective shielding materials with an efficiency comparable to traditional shielding materials (lead and concrete). The addition of the Bi_2_O_3_ compound to the composition of ceramics is due to the possibility of reducing the temperature of phase transformations, as well as increasing the solubility of the components during sintering. The choice of the PbO compound as a dopant is due to its physical properties, as well as its high density, which makes it possible to increase gamma radiation absorption efficiency without affecting the optical transmission capacity. The addition of MoO_3_, SiO_2_ compounds to the composition is due to their strength properties, which consist in increasing the resistance of ceramics to external influences [41,42].

For notation convenience, in the further description of the results obtained, the abbreviated designation of the objects of study was introduced depending on the PbO dopant concentration. Table 1 presents data on the molar ratio of ceramic components as well as their abbreviated name.

**Figure 1:**
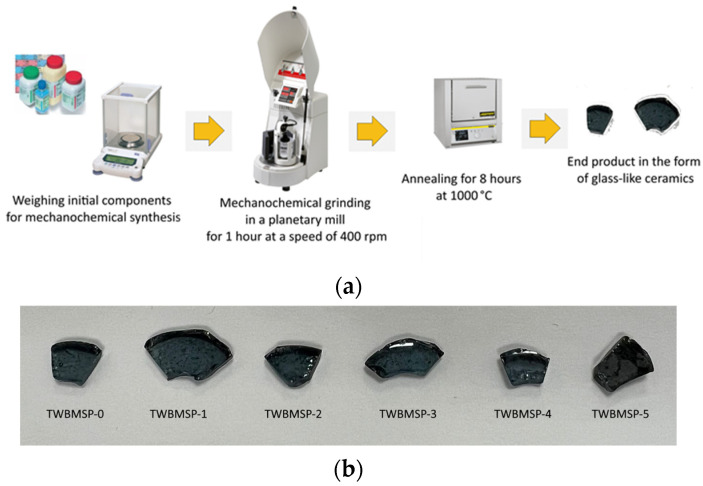
(**a**) Scheme for obtaining glass-like ceramics using mechanochemical grinding method and further thermal annealing; (**b**) Images of composite glass-like ceramics depending on PbO concentration.

After weighing in a given molar ratio, the obtained mixtures were subjected to mechanochemical grinding in a Pulverisette 6 classic line planetary mill (Fritsch, Berlin, Germany). Grinding was conducted at a grinding speed of 400 rpm for 1 h. After grinding, the obtained mixtures were subjected to thermal sintering at a temperature of 1000 °C in a muffle furnace (Nabertherm GmbH, Lilienthal, Germany). Sintering was carried out for 8 h, AT A heating rate of 10 °C/min, heating to 1000 °C was carried out in 100 min. After exposure for 8 h at a temperature of 1000 °C, the samples were cooled down together with the muffle furnace to reach room temperature within 10–15 h. The samples were sintered in a muffle furnace in air. The obtained ground samples were placed in alundum crucibles, after which they were placed in a furnace and subjected to sintering. The sintering process itself was carried out without applying external pressure. The choice of sintering temperature is because of the initialization of the TeO_2_ and WO_3_ compound melts formation processes, which lead to the formation of vitreous ceramics. The scheme for obtaining glass-like ceramics is shown in Figure 1a.

Figure 1b represents images of the obtained composite glass-like ceramics after sintering in crucibles to reflect the degree of transparency and appearance.

As is evident from the data presented, in the synthesized glasses, with the PbO concentration growth, the formation of small inclusions is observed, as well as a slight darkening of the glasses, which may be associated with a change in the optical density or reflectivity of the composite glass-like ceramics.

The morphological features of the synthesized ceramics were analyzed using scanning electron microscopy using a Hitachi TM 3030 microscope (Hitachi, Tokyo, Japan) equipped with an attachment for energy dispersive analysis and determining the uniformity of the elemental distribution in the structure.

Analysis of the structural ordering and phase transformation processes depending on the PbO dopant concentration was conducted using the X-ray diffraction method implemented on a D8 Advance Eco powder diffractometer (Bruker, Berlin, Germany). The diffraction patterns were obtained using the Bragg–Brentano geometry in the angular range of 2θ = 25 − 90°. The ICSD database was used to analyze phase inclusions.

The dislocation density was calculated based on the data obtained by X-ray diffraction, as well as changes in grain sizes. To estimate the dislocation density (*δ*), the expression *δ* = 1/*D*^2^ was used, which reflects the relation among the dislocation density and grain sizes (*D*).

The optical properties of the synthesized ceramics were studied by analyzing the obtained UV-Vis spectra with their subsequent processing and calculation of optical characteristics. A Jena Specord-250 spectrophotometer (Analytic Jena, W 11th St, Upland, CA, USA) was used for measurements.

The mechanical properties of the synthesized samples were studied using two methods for determining the strength parameters. The hardness was determined by indentation using a LECO 700M microhardness tester, a Vickers pyramid was used as an indenter, the pressure on the indenter was 100 N. Based on the change in indenter prints, the values of hardness and hardening of ceramics were determined depending on the dopant concentration. The resistance to cracking and chipping was determined by the method of single compression at a constant compression rate of 0.1 mm/min.

Determination of the shielding characteristics of the studied ceramics depending on the change in the concentration of the PbO dopant was carried out using a standard technique, which includes the following scheme. At 10 cm from the NaI detector used as a gamma-ray detector, a gamma-ray source (Co^57^ with a gamma-ray energy of 130 keV, Cs^137^ with a gamma-ray energy of 660 keV, Na^22^ with a gamma-ray energy of 1270 keV) is placed, enclosed in a lead container. A protective shield made of synthesized ceramics is placed in front of the source, after which the transmitted radiation intensity is measured for a given period of time, the value of which is used to determine the shielding parameters.

## 3. Results and Discussion

**Figure 2:**
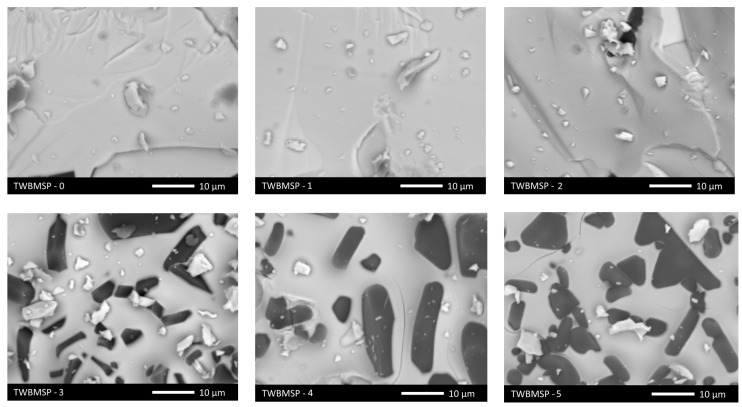
SEM images of the morphology of the synthesized ceramics depending on the concentration of the PbO dopant.

Figure 2 shows the SEM images of the studied samples depending on the concentration of the PbO dopant in the composition of the ceramics.

As can be seen from the data presented, with a growth in the PbO dopant concentration to 0.10 mol, the formation of grains differing in their structure from the general form of the obtained samples is observed. These grains are elongated, diamond-shaped, or rectangular inclusions embedded in the bulk of the ceramics. At the same time, an increase in the PbO dopant concentration results in an increment in the number of these grains, as well as their enlargement, followed by the formation of agglomerate inclusions in the composition. The presence of such structures may be due to the effect of the formation of impurity phases, and the shape of the arrangement of these grains indicates the formation of structures in the form of an interstitial or substitutional solid solution.

**Figure 3:**
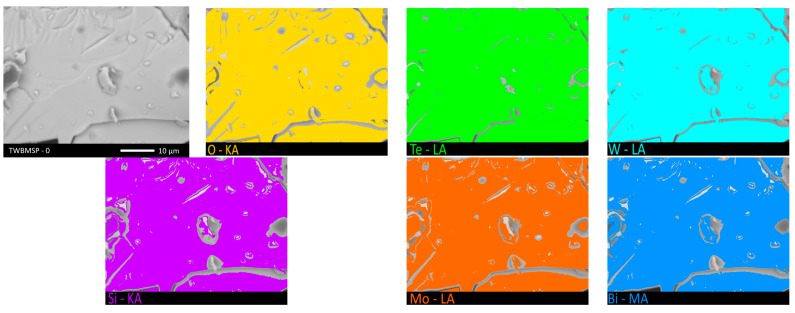

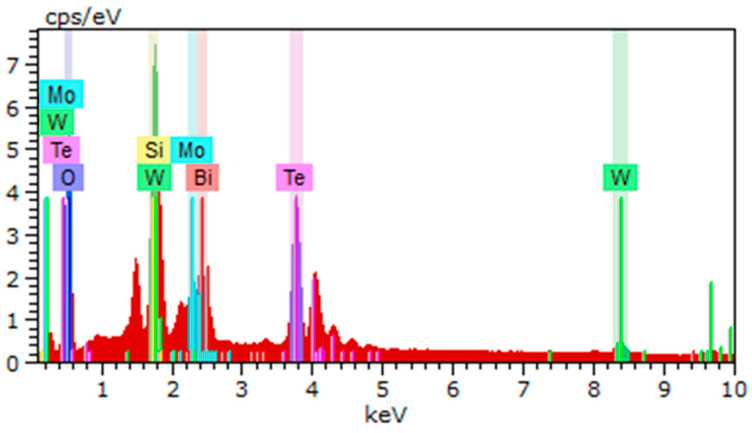
Mapping results for TWBMSP-0.

**Figure 4:**
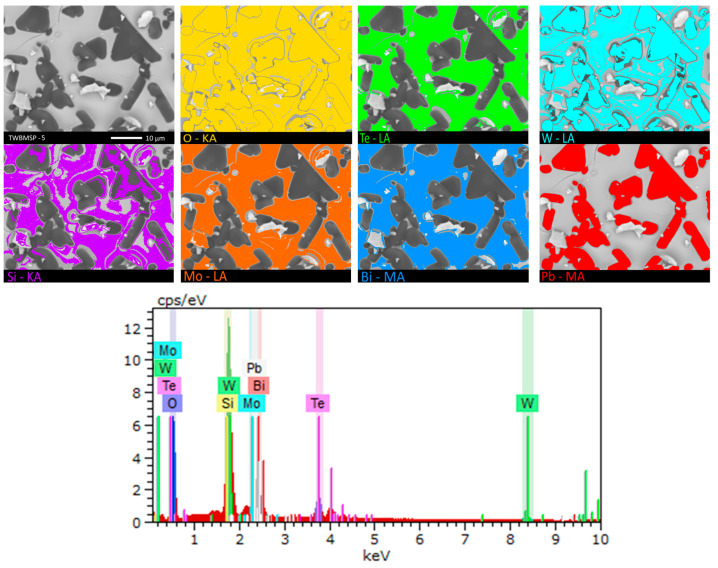
Mapping results for TWBMSP-5.

To estimate the elemental composition of these inclusions, the energy dispersive analysis method in the form of mapping was applied. Results of the studies are shown in Figure 3 and Figure 4. Results of mapping were performed for samples not containing the PbO dopant and with the maximum concentration for which, according to the data of morphological studies, the formation of agglomerates of grain inclusions is observed.

As is evident from the obtained mapping data, in the case of the original samples not doped with PbO, the distribution of elements is equiprobable and isotropic in the structure of ceramics, while for the doped samples, the grains formed, according to the mapping data, contain large amounts of tungsten, oxygen, and lead, as well as an insignificant the amount of silicon. In this regard, it can be concluded that the grains formed as a result of PbO doping are inclusions of another phase or structural formations with a structure different from the general composition.

**Figure 5:**
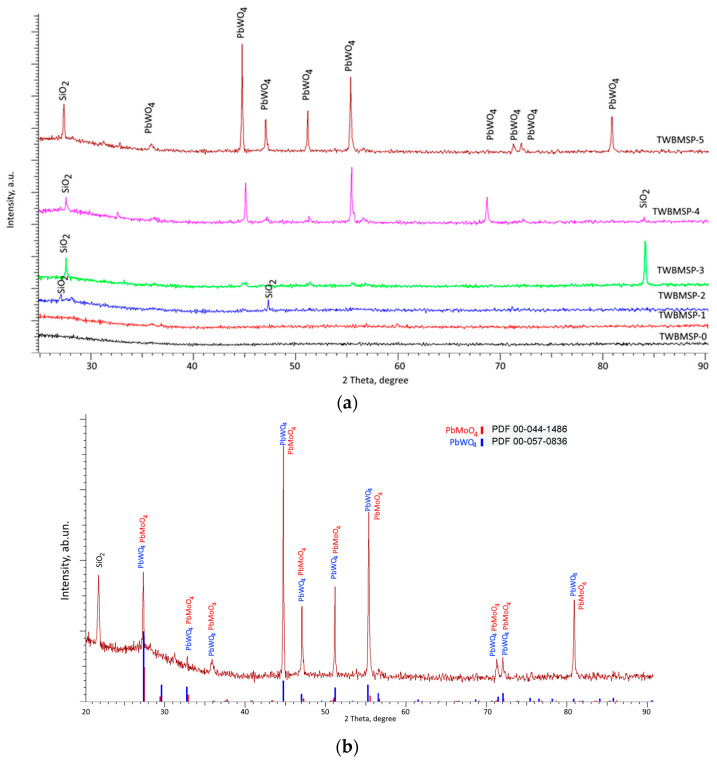
(**a**) X-ray diffraction patterns of the studied samples as a function of the PbO dopant concentration; (**b**) The results of X-ray diffraction of the TWBMSP-5 sample, for which the presence of diffraction reflections for the PbWO_4_ and PbMoO_4_ phases was observed.

Figure 5 shows X-ray diffraction patterns of the studied samples, obtained by powder diffraction. Diffraction patterns were obtained depending on the PbO dopant concentration in the samples. The overall view of the observed changes indicates the processes of phase and structural transformations depending on the dopant concentration, which are expressed in changes in the diffraction patterns, as well as the structural ordering degree.

Analysis of the obtained diffraction patterns showed the following. In the case of TWBMSP-0 samples, in which the PbO dopant is absent, no obvious low-intensity or strongly broadened diffraction reflections were observed. Such a diffraction pattern is characteristic of amorphous-like or highly disordered structures, which indicates that the nature of the TWBMSP-0 samples is amorphous and typical of glasses obtained from oxide compounds at high sintering temperatures. A similar nature of telluride glasses was observed in several works, according to which sintering at temperatures above 700–800 °C results in amorphization of the glass structure.

A similar diffraction pattern was also observed for the TWBMSP-1 sample, for which the PbO dopant concentration was 0.05 mol. In this case, according to X-ray diffraction data, a low dopant concentration does not significantly affect the increase in the structural ordering degree and the appearance of phase inclusions.

In the case of TWBMSP-2 samples for which the PbO dopant concentration was 0.10 mol, the formation of low-intensity reflections in the region of 2θ = 23.0 − 23.6° and 2θ = 47.0 − 48.0°, which are characteristic of inclusions of tetragonal SiO_2_, is observed. The appearance of these reflections indicates that during sintering, reactions occur leading to the formation of structurally ordered inclusions of SiO_2_ in the composition of the samples. A similar picture is also observed for sample TWBMSP-3, on the diffraction pattern of which diffraction reflections characteristic of SiO_2_ are also observed, while the appearance of an additional reflection in the 2θ = 84.0 − 85.0° region, characteristic of SiO_2_, indicates not only the structural ordering of these inclusions, but also the formation of a preferred textural orientation in them, characteristic of the tetragonal structures of SiO_2_. The formation of such inclusions depending on the PbO dopant concentration can be explained by the influence of the dopant on the sintering of samples, followed by the formation of structurally ordered inclusions from poorly soluble compounds.

According to a detailed analysis of the X-ray diffraction pattern of the TWBMSP-5 sample, presented in Figure 5b, it was revealed that the observed reflections can be compared to two phases PbWO_4_ and PbMoO_4_ with a tetragonal type of crystal lattice and space symmetry group I41/a(88). According to X-ray phase analysis, the probability of coincidence of the position and intensity of the main reflections observed with the position of the lines of reference values from the PDF-2 (2016) database is more than 80% for the PbWO_4_ phase, while for the PbMoO_4_ phase it is no more than 50–60%. In this case, these phases represent isostructural compounds of the scheelite type, which can represent a solid solution. However, an analysis of the density of the obtained ceramics measured using the Archimedes method showed that an increase in the PbO concentration from 0.20 mol to 0.25 mol results in a rise in density from 6.87 to 7.91 g/cm^3^. Such a change in density may be due to the dominance of the PbWO4 phase in glasses, since the theoretical value of the density for this phase is more than 8.41 g/cm^3^.

As is known, the PbWO_4_ compound, which has a symmetrical scheelite structure, is most likely to contain Pb^2+^ ions, the presence of which results into a reduction of the interaction of the 6s orbital with oxygen, thereby reducing its role in the formation of crystal chemical bonds. In this case, the relativistic contraction of the 6s orbital leads to the stabilization of the symmetry of the scheelite structure [43,44,45,46].

In accordance with the presented X-ray diffraction data, a growth in the PbO concentration in the composition of vitreous ceramics results into the formation of a tetragonal PbWO_4_ phase in the structure, the appearance of which is fixed at a PbO concentration of 0.1 mol, and with a further increase in the PbO content, a structural ordering of these inclusions is observed, which is expressed in the formation of well-defined diffraction patterns reflexes.

According to the Gibbs free energy (∆G) estimate for the chemical reaction PbO + WO_3_ → PbWO_4_, the value ∆G = −57.32 kJ/mol, which is much less than 0 (∆G < 0), thus indicating that this reaction proceeds freely at selected synthesis conditions.

Calculations of ∆G carried out for all other chemical compounds significantly exceed 0, which indicates the impossibility of such reactions with the possibility of the complex oxide formation, and confirms the X-ray phase analysis data, according to which no reflections were found for other phases.

In the case of a PbO dopant concentration of 0.20–0.25 mol, the diffraction patterns show the appearance of diffraction reflections characteristic of the tetragonal phases of PbWO_4_ and SiO_2_, and the shape of the diffraction reflections, as well as their intensity, change with the dopant concentration rise from 0.20 to 0.25 mol. The formation of these inclusions indicates the processes of phase transformations as a consequence of sintering, which is characteristic of the formation of highly ordered structural inclusions in the form of tetragonal phases. While for samples with a dopant concentration of 25 mol, the PbWO_4_ phase is dominant of the two phases. No other structural inclusions were found in the sample’s composition.

Thus, by analyzing the obtained variations in the diffraction patterns of the samples as a function of the dopant concentration, we can conclude that a rise in the PbO dopant concentration above 0.10 mol leads to the initialization of the processes of phase transformations and structural ordering related to the formation of inclusions of tetragonal PbWO_4_ and SiO_2_ phases in the amorphous matrix. The obtained results of X-ray phase analysis confirm the data of morphological studies and elemental analysis that the formed grain inclusions have a structure different from the general composition and are PbWO_4_ and SiO_2_ inclusions.

**Figure 6:**
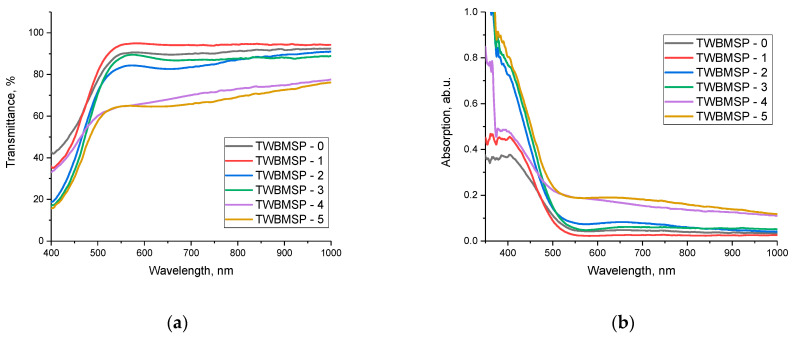
Results of the optical properties of samples under study: of the optical properties of the samples under study: (**a**) optical transmission spectra; (**b**) optical absorption spectra.

The study of the optical properties of the synthesized ceramics was carried out by analyzing the obtained transmission and absorption spectra in the UV-Vis range, which makes it possible to determine the alteration in the band gap and to establish the dependences of the influence of the formation of the PbWO_4_ and SiO_2_ phases on the transmission and absorption abilities. The results of changing the optical spectra are represented in Figure 6.

The overall appearance of the presented dependences of the optical transmission and absorption spectra indicates a variation in the transmission and absorptivity of the synthesized ceramics as a function of the variation in the PbO dopant concentration. An analysis of the transmission spectra indicates that the main observed alterations are associated with two factors. The first factor of variations is related to an alteration in the transmission value in the range of 400–1000 nm, a decrease in which may be related to the formation of PbWO_4_ and SiO_2_ inclusions in the structure, leading to an increase in optical absorption and a change in the optical density of ceramics. The second factor of changes is associated with a change in the fundamental absorption edge, the alteration of which is due to a variation in the electron density and a change in the band gap.

In the case of absorption spectra, the formation of PbWO_4_ and SiO_2_ inclusions results into a growth in the absorption capacity in the region of 350–550 nm, which shows a variation in the optical properties of ceramics.

**Figure 7:**
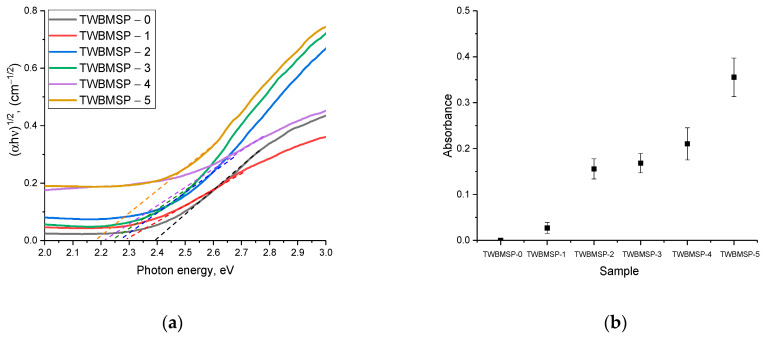
Results of the optical properties of samples under study: (**a**) Tauc’ plots; (**b**) Optical density results.

Based on the spectra obtained, Tauc plots were constructed, reflecting the variation in the band gap, and the analysis of the transmission and absorption spectra was used to estimate the optical density of the ceramics (absorbance), the change in which reflects the alteration in optical properties. Results of the variation in these values are shown in Figure 7.

As is evident from the data presented, a change in the dopant concentration leads to a shift of the fundamental absorption edge to the region with lower energies, which indicates a reduction in the band gap, the results of which are presented in Table 2.

Using Formulas (1)–(5), changes in the optical characteristics obtained from the analysis of the experimental transmission and absorption spectra were evaluated.

The refractive index (*n^optical^*) was determined using Formula (1):

(1) $$\frac{\left[{\left(n^{optical}\right)}^{2}-1\right]}{\left[{\left(n^{optical}\right)}^{2}+2\right]}=1-\sqrt{\frac{E_{g}}{20}}$$

The optical transmission (*T^optical^*) and refractive loss (*R^loss^*) were calculated using Equations (2) and (3):

(2) $$T^{optical}=\frac{2\left(n^{optical}\right)}{{\left(n^{optical}\right)}^{2}+1}$$

(3) $$R^{loss}={\left(\frac{\left(n^{optical}\right)-1}{\left(n^{optical}\right)+1}\right)}^{2}$$

Molar refraction (*R^molar^*) was determined by Formula (4):

(4) $$R^{molar}=\left(\frac{{\left(n^{optical}\right)}^{2}-1}{{\left(n^{optical}\right)}^{2}+2}\right)V^{molar}$$

The metallization criterion (*M*) was determined using Equation (5):

(5) $$M=1-\frac{R^{molar}}{V^{molar}}$$

It is important to note that the variation in the optical density value is most pronounced in the case when phase transformations are observed in the structure associated with the formation of ordered structural inclusions, the presence of which reduces the concentration of the amorphous fraction. The formation of structurally ordered inclusions in the form of PbWO_4_ and SiO_2_ grains results in the appearance of additional grain boundaries that increase absorption and create defects for the passage of light, thereby changing the optical properties. At the same time, an alteration in the phase composition of ceramics as a consequence of the formation of structurally ordered inclusions results in a rise in the metallization factor and a growth in the refractive index of ceramics.

One of the important characteristics of shielding materials used for protection against the adverse effects of ionizing radiation, in addition to the shielding absorbing characteristics themselves, are indicators of resistance to external influences, including mechanical shock or damage. These indicators make it possible to determine the level of applicability of shielding materials when used as a basis for protection against ionizing radiation under conditions not only of an increased background radiation, but also of possible external influences. While in case of brittleness of protective materials, under external pressure or impacts, their destruction may occur, which will result in the material destruction, as well as the shielding efficiency reduction in the event of partial destruction of the protective material. In general, the strength properties of materials are evaluated in terms of resistance to external influences and crack resistance, parameters that make it possible to evaluate the mechanical strength of the material. Moreover, as is known, in most cases, a change in the structure of materials, including the transition from an amorphous state to a crystalline state, can be accompanied by a change in strength characteristics. Moreover, a prominent role in this case is played by the effect of the dislocation density in the material, as well as the density of the material itself, which changes in the case of a change in the phase composition.

**Figure 8:**
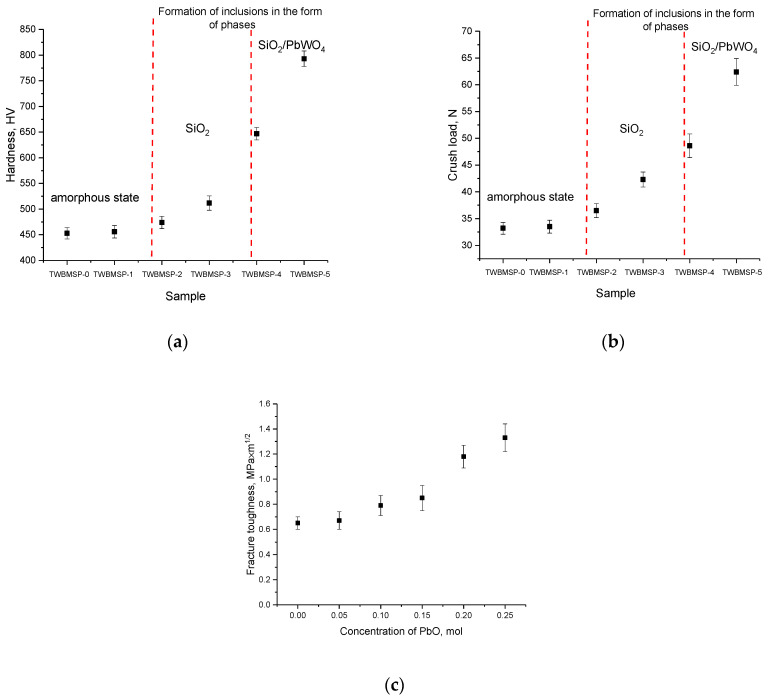
Results of the change in mechanical characteristics: (**a**) hardness data depending on the sample type; (**b**) single compression data; (**c**) fracture toughness data.

Figure 8 demonstrates the results of variations in the indicators of strength characteristics: hardness and resistance to single compression, parameters that characterize the alteration in the mechanical properties of ceramics as a function of the dopant concentration. As is evident from the data presented, in the case when the glass-like amorphous phase predominates in the obtained ceramics (according to X-ray phase analysis), the hardness and crack resistance are low, which indicates a low resistance to mechanical stress and cracking during compression or external impacts. The formation of the SiO_2_ phase in the structure of inclusions results in a slight increase in mechanical parameters, while the trend of changes has a rather pronounced dependence on changes in the phase state and the transition from an amorphous-like structure to an ordered one. The formation of PbWO_4_ inclusions in the structure of ceramics in the form of grains with a high structural ordering degree results in a sharp increase in mechanical parameters, which shows a rise in resistance to external influences and a growth in resistance to cracking and chipping under external pressures and compression.

Figure 8c presents the results of assessing the variation in the fracture toughness value, which makes it possible to assess the resistance of the material to the propagation of cracks under compression. The overall appearance of the established trend in the variation in resistance to cracking when changing the type of ceramics from an amorphous state to being present in the composition of crystallized inclusions reflects a growth in resistance to the formation and further propagation of cracks.

**Figure 9:**
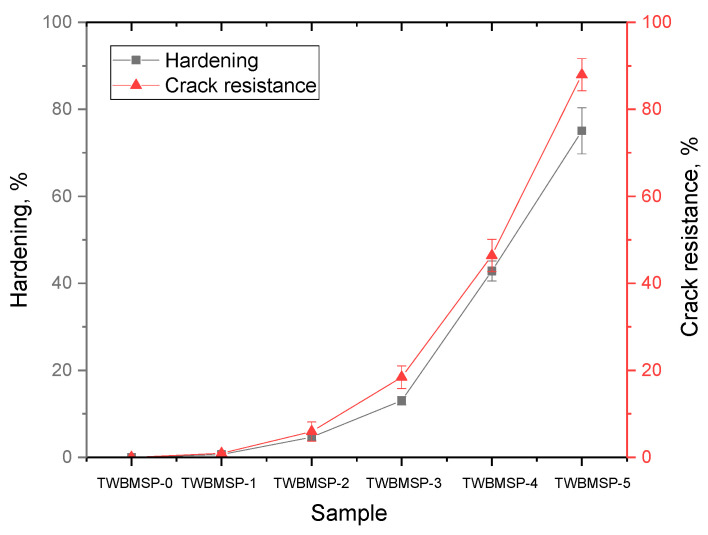
Results of variations in the strengthening characteristics of the synthesized ceramics.

Figure 9 demonstrates the data on variations in the indicators of ceramic hardening and increase in crack resistance depending on the sample type, which reflect the alteration in the mechanical properties of the synthesized ceramics.

As is evident from the data provided, the greatest efficiency of changing the mechanical properties associated with hardening and increasing resistance to external influences is observed during the formation of PbWO_4_ inclusions in the structure, as well as transformations of the “amorphous glass” → “glass-like ceramics with well-ordered phase inclusions” type, which result into a growth of resistance to external influences. The hardening effect can be significantly affected by morphological changes associated with the formation in the structure of ceramics of inclusions in the form of grains, which are PbWO_4_ particles. The presence of these inclusions results in a change in the structural properties, phase transformations, as well as the formation of additional grain boundaries, leading to the appearance of boundary effects near which dislocations accumulate and prevent crack propagation under mechanical stress.

**Figure 10:**
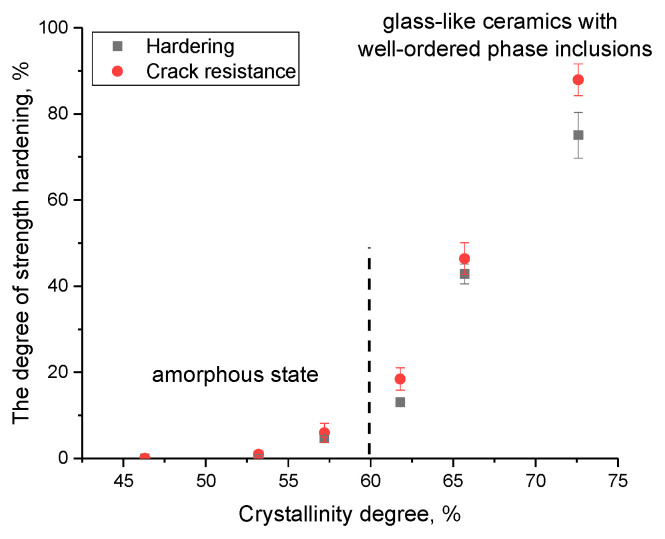
Dependence of the change in strength characteristics on the structural ordering degree.

To compare and evaluate the effect of a change in the structural ordering degree because of the formation of PbWO_4_ inclusions on the strengthening of ceramics, the following dependences were plotted, shown in Figure 10. These dependences reflect the change in the ratio of the amorphous and ordered fractions in ceramics on the variation in strength characteristics. The ratio of the contributions of the amorphous and ordered fractions associated with the formation of PbWO_4_ grains was estimated via a comparative analysis of the reflection areas using the following Formula (6):

(6) $$Crystallinity=\frac{S_{cr}}{S_{cr}+S_{am}}\times 100\%,$$

where *S_cr_* are the areas of diffraction reflections, and *S_am_* is the area characteristic of disordered regions or an amorphous halo.

**Figure 11:**
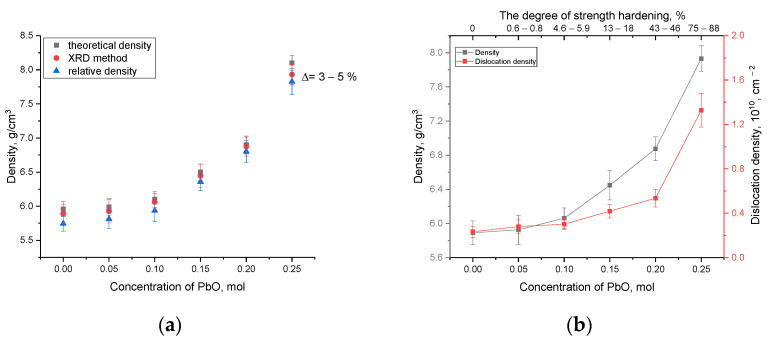
(**a**) The results of variations in the density of ceramics depending on the PbO dopant concentration; (**b**) Results of variations in density and dislocation density depending on the PbO dopant concentration.

As is evident from the data presented, the strengthening of ceramics is observed with an increase in the structural ordering degree above 60%, which means that the proportion of formed grains in the structure of ceramics prevails over the amorphous component. It is also worthwhile to note that the most pronounced change in strength characteristics is manifested in a crack resistance growth, which increases due to the formation of a grain structure that prevents the propagation of cracks under external influence. At the same time, as is known, the formed PbWO_4_ grains are inorganic compounds of lead and tungsten with a density of more than 8.2 g/cm^2^, which makes them one of the promising materials for the shielding and absorption of gamma radiation. In turn, the effect of strengthening ceramics can also be due to the effect of a change in the density of ceramics when grains dominate in the PbWO_4_ structure. Figure 11a represents the results of a comparative analysis of changes in the density of the obtained glass-like ceramics. Density estimation was carried out by two methods: X-ray phase analysis based on changes in structural parameters and the standard method of Archimedes. For comparison, the results of a theoretical assessment of the density of ceramics based on the used molar ratios of the initial components are presented. As is evident from the data presented, the density results obtained by various methods have close values, and the difference in the form of a deviation from the theoretical values is no more than 3–5%. The results of changing the density of ceramics, and the dislocation density calculated on the basis of data on changes in grain sizes, are shown in Figure 11b. These parameters can also have a great effect on the strengthening of ceramics.

As is evident from the data presented, the formation of the PbWO_4_ phase in the composition of ceramics, as well as an increase in the structural ordering degree, leads to a rise in the density of ceramics and a variation in their dislocation density, which has a great impact on the strengthening of, as well as alterations in the mechanical properties of ceramics.

One of the most promising areas of application of these ceramics is their use as shielding materials for protection against the adverse effects of ionizing radiation and absorption of gamma rays in order to reduce their intensity. At the same time, the change in the phase composition of ceramics, and their density due to the formation of inclusions in the form of PbWO_4_ grains, can have a dramatic impact on the shielding and absorbing characteristics, due to the fact that the formed structures of lead tungsten have high efficiency in absorbing high-energy radiation, including gamma radiation, which is actively used in scintillation technology in registration and spectroscopy of ionizing radiation.

**Figure 12:**
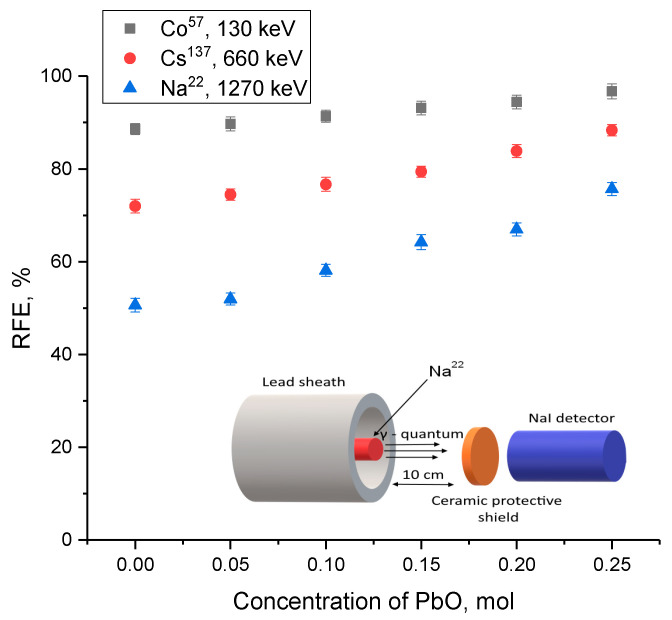
Results of determining the efficiency of gamma radiation intensity reduction when synthesized ceramics are used as shielding materials (the inset to the figure shows a schematic representation of experiments on shielding gamma radiation).

An evaluation of the gamma radiation shielding efficiency with the energy range of gamma radiation of 130–1270 keV is shown in Figure 12. Three sources of gamma quanta were chosen to conduct experiments on evaluating the shielding characteristics: Co^57^ with an energy of 130 keV, which makes it possible to simulate gamma quanta interaction processes by the photoelectric effect mechanism; Cs^137^ with an energy of 660 keV, which makes it possible to evaluate the mechanisms of gamma radiation interaction with matter according to the Compton effect type, with a subsequent change in their wavelength; Na^22^ with the energy of emitted gamma-quanta of 1270 keV, the main interaction mechanisms of which are the formation of electron-positron pairs. The intensity reduction efficiency (*RFE*) value was estimated using Formula (7):

(7) $$RFE=\left(1-\frac{I}{I_{0}}\right)\times 100\%$$

where *I* and *I*_0_ are the gamma radiation intensity values recorded by the detector with and without a shield.

The overall appearance of the presented dependences indicates a positive effect of the alteration in gamma radiation shielding efficiency with an increase in the PbO dopant concentration. While the shielding efficiency of gamma radiation with an energy of 130 keV for all cases is more than 85%, which indicates a high shielding efficiency, it should also be noted that in the case of shielding gamma rays with an energy of 130 keV, a change in dopant concentration, leading to the formation of PbWO_4_ inclusions, the change in efficiency is no more than 5–10% with an increase in concentration and phase transformations. Such a change may be due to the fact that almost complete absorption occurs for these low-energy gamma quanta, and due to the interaction mechanisms, a variation in the phase composition does not have a particularly large effect on the absorption efficiency. At an energy of gamma rays of 660 keV, which are characterized by interaction mechanisms associated with the Compton effect, an increase in the concentration of the PbO dopant results in a more pronounced alteration in the absorption efficiency, which indicates that a variation in the phase composition of ceramics has a greater effect on the absorbing ability.

A similar situation, with a more pronounced increase in the absorption efficiency, is also observed during shielding of gamma rays with an energy of 1270 keV, which are characterized by the formation of electron-positron pairs. In that case, a rise in the contribution of the PbWO_4_ phase results in a sharp increase in the shielding efficiency. This effect is due to the fact that lead tungstate structures have a high absorbing capacity for high-energy gamma rays due to the large nuclear charge and high Z_eff_ value.

**Figure 13:**
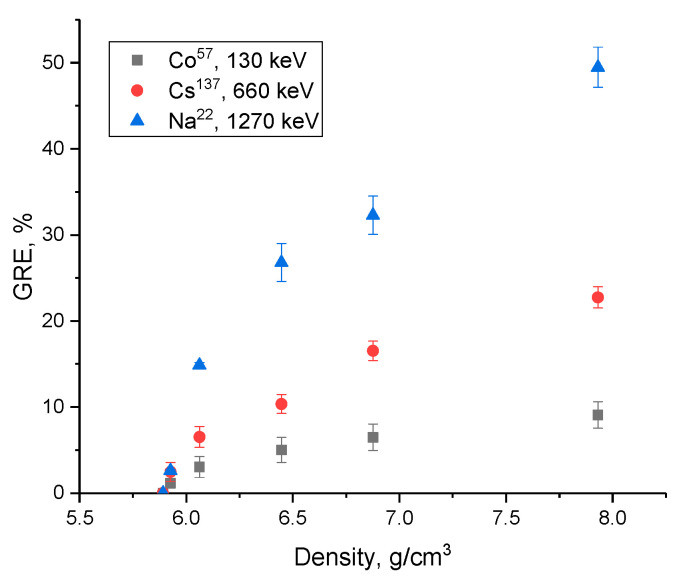
Comparative analysis of evaluation of the shielding efficiency of ceramics when density changes with the dopant concentration increase.

Figure 13 presents the results of a comparative analysis of the variation in the shielding efficiency depending on the density of the synthesized ceramics with an increase in the PbO dopant concentration, leading to phase transformations of the “amorphous glass” → “glass-like ceramics with well-ordered phase inclusions” type in comparison with the undoped sample. Shielding efficiency evaluation (*GRE*) was carried out using Formula (8):

(8) $$GRE=\left(\frac{RFE_{i}-RFE_{0}}{RFE_{0}}\right)\times 100\%$$

where *RFE*_0_ and *RFE_i_* are the shielding efficiency values for samples without dopant and containing PbO dopant.

Analysis of the estimation of the shielding and absorption efficiency as a function of density change with a growth in the dopant concentration and, as a result, the formation of ceramics with well-ordered phase inclusions in the form of PbWO_4_ grains, showed that the obtained dependences for shielding gamma-quanta with energies of 130 keV and 660 keV can be described by an exponential relationship reflecting the absorption efficiency saturation effect with increasing density of samples. In the case of shielding gamma rays with an energy of 1270 keV, the dependence of the alteration in efficiency on the change in the density of ceramics has a different character than in the case of shielding of gamma radiation in the range of 130–660 keV. The change in density due to the formation of PbWO_4_ inclusions results in a sharp growth in efficiency compared to undoped samples by more than 25–30%, and the maximum increase in efficiency is more than 50% compared to undoped samples.

**Figure 14:**
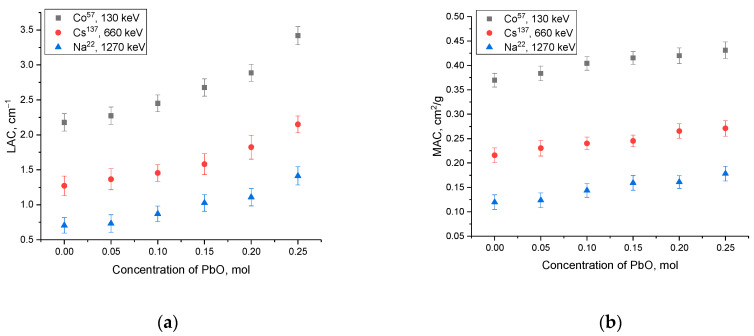
Shielding performance results: (**a**) linear absorption coefficient change data; (**b**) mass absorption coefficient change data.

Figure 14 shows the calculation results of the linear and mass absorption coefficients, which are used in most cases to design shielding coatings and determine the most effective absorption layer to use for shielding.

Linear and mass coefficients were evaluated using Formulas (9) and (10).

(9) μ=lnI0Id

(10) $$\mu_{m}=\frac{\mu }{\rho }$$

where *d* is the thickness and *ρ* is the density of the samples under study.

As is evident from the data presented, a growth in the concentration of the PbO dopant in the composition of ceramics results into a rise in the absorption efficiency and a greater decrease in the intensity of gamma radiation. In this case, the most pronounced change in the efficiency of shielding characteristics in the case of a change in the linear and mass absorption coefficient is observed when shielding gamma rays with an energy of 130–660 keV, for which the absorption efficiency changes by more than 1.1–1.5 times with a change in the phase composition of ceramics. Thus, the obtained dependences of the change in absorption characteristics indicate the high efficiency of using these types of ceramics as shielding materials.

## 4. Conclusions

Following the X-ray diffraction method, it was revealed that a rise in the PbO dopant concentration results in the initialization of phase transformation processes of the “amorphous glass” → “amorphous glass with structurally ordered inclusions” → “glass-like ceramics with well-ordered phase inclusions” type. During analysis of the strength properties of the synthesized ceramics, it was revealed that the formation of PbWO_4_ in the structure results in an increase in the stability of the strength characteristics by 70–80% compared to the original samples and, at the same time, crack resistance increases. Based on the studies performed to evaluate gamma radiation shielding efficiency, it can be concluded that a change in the structure of the synthesized ceramics with the formation of PbWO_4_ inclusions results in a rise in the efficiency of the absorption of high-energy gamma rays, which is because of the effects of the alteration in the Z_eff_ and the density of ceramics.

## Figures and Tables

**Table 1:** Designation of samples.

Sample	(1 − x)TeO_2_ − 0.2WO_3_ − 0.1Bi_2_O_3_ − 0.1MoO_3_ − 0.1SiO_2_ − xPbO					
	TeO_2_, mol	WO_3_, mol	Bi_2_O_3_, mol	MoO_3_, mol	SiO_2_, mol	PbO, mol
TWBMSP-0	0.50	0.20	0.10	0.10	0.10	0
TWBMSP-1	0.45					0.05
TWBMSP-2	0.40					0.10
TWBMSP-3	0.35					0.15
TWBMSP-4	0.30					0.20
TWBMSP-5	0.25					0.25

**Table 2:** Optical characteristics of the studied ceramics.

Parameter	Sample					
	TWBMSP–0	TWBMSP–1	TWBMSP–2	TWBMSP–3	TWBMSP–4	TWBMSP–5
*Band gap* (*E_g_*), *eV*	2.359	2.290	2.267	2.241	2.218	2.183
*n_optical_*	2.595	2.620	2.629	2.638	2.647	2.661
*T_optical_*	0.197	0.201	0.202	0.203	0.204	0.206
*R_loss_*	0.671	0.667	0.664	0.662	0.661	0.658
*M*	0.342	0.347	0.364	0.421	0.475	0.532

## Data Availability

Not applicable.
